# Effects of a feed supplement, containing undenatured type II collagen (UC II®) and Boswellia Serrata, in the management of mild/moderate mobility disorders in dogs: A randomized, double-blind, placebo controlled, cross-over study

**DOI:** 10.1371/journal.pone.0305697

**Published:** 2024-10-30

**Authors:** Marzia Stabile, Laura Fracassi, Luca Lacitignola, Elena Garcia-Pedraza, Chiara Roberta Girelli, Crescenza Calculli, Angela Maria D’Uggento, Nunziata Ribecco, Antonio Crovace, Francesco Paolo Fanizzi, Francesco Staffieri

**Affiliations:** 1 Dipartimento di Medicina di Precisione e Rigenerativa e Area Jonica (Di.Me.Pre-J), Università Degli Studi di Bari, Valenzano, Bari; 2 Vetoquinol SA, Paris, France; 3 Dipartimento di Scienze e Tecnologie Biologiche e Ambientali (DiSTeBA), Università del Salento, Lecce, Italia; 4 Dipartimento di Economia e Finanza, Università degli Studi di Bari, Bari, Italia; Sheikh Hasina National Institute of Burn & Plastic Surgery, BANGLADESH

## Abstract

This study was designed as a randomized, placebo-controlled, double-blinded, cross-over trial performed to investigate the effects of a dietary supplement containing undenatured type II collagen (UCII^®^) and *Boswellia Serrata* on mobility, pain and joint metabolism in mild moderate osteoarthritis (OA) in dogs. A total of 60 dogs with mobility problems were evaluated and enrolled in the study. Seventeen of these dogs with mild/moderate OA were randomized to receive the product A (UCII^®^ + *Boswellia Serrata* supplement–UCII^®^-BW) or product B (Placebo -PL), 1 chew per day for 8 weeks by oral route, and repeated in a crossover design after 4 weeks of washout period. All the subjects had veterinary evaluations during the trial and owners were requested to fill out a questionnaire on mobility impairment using the Liverpool Osteoarthritis in dogs scale (L.O.A.D.) at each time of the study. Objective tools were used to assess mobility, activity, and pain. Metabolomic analysis was performed on synovial fluid of most affected joint at the beginning and the end of the study. The results proved that UCII^®^+*Boswellia serrata* supplemented group over a period of eight weeks results in an improvement of mobility impairment, already at 4 weeks of administration, according to the owner´s evaluation. In contrast, its absence increased the risk of OA crisis and decreased the pain threshold on the most affected joint. Furthermore, the synovial fluid metabolic profile showed moderate differences between the beginning and the end of the supplementation period, with a particular influence associated to the time of UCII^®^-BW administration.

## Introduction

Canine osteoarthritis (OA) is a common form of joint pain caused by persistent low-grade inflammation and characterised by bone remodelling, synovitis and articular cartilage degeneration [1–3].

Genetics, trauma, joint incongruity, dysplasia and also, metabolic conditions, are just a few of the many variables that can affect the onset and progression of this disease [4,5].

The pain caused by OA leads to a variety of signs such as stiffness, lameness, refusal to jump, run or use stairs, reluctance to get up, restlessness, lack of enthusiasm to walk or play, irritability or aggression [4,6]. All of these signs are easily recognised by the owner and can help the clinician make the diagnosis, which is confirmed by orthopaedic and radiographic examination [4,7,8]. In recent years, the crucial role of the owner in the management of this condition has been fully acknowledged, and new tools have been developed for staging the disease [9,10] and for assessing pain and mobility impairment [11–14], including both owner and clinician assessment. Despite many efforts to improve the detection and early diagnosis of OA, the main problem remains the lack of a definitive cure. Therefore, the clinician’s main objective remains to relieve pain, protect the joint from disease progression, improve mobility and quality of life. The strategies used to treat OA are based on a multimodal approach that includes pharmacological and non-pharmacological treatment. To preserve joint condition, the use of oral dietary supplements are suggested in addition to anti-inflammatory drugs and treatments to reduce persistent pain, such as monoclonal antibody anti-NGF, gabapentin, amantadine, and tramadol [15–17]. Among the oral administration products used for OA in veterinary care, a new formulation, including undenatured type II collagen (UC II^®^) + *Boswellia Serrata* has been recently commercialized. These feed supplements might reduce the inflammatory state and oxidative stress on the cellular level, acting through different mechanisms of action. In particular, this new formulation combines the immunomodulatory effects of undenatured type II collagen combined with the natural benefits of the effects of *Boswellia serrata* in the inflammatory cascade [18].

Undenatured type II collagen acts through the interaction of the active epitopes of type II collagen with Peyer’s patches by a mechanism known as oral tolerance, promoting the conversion of naive T cells into T regulatory (Treg) cells and the release of their anti-inflammatory cytokines into the joint, such as transforming growth factor-beta (TGF-beta), interleukin-4 (IL-4) and interleukin-10 (IL-10) [17]; while *Boswellia serrata* extracts appear to act by inhibiting leukotriene synthesis, production of pro-inflammatory cytokines, reduction of oxygen free radicals and inhibition of the complement system [19,20]. Thus, both substances may contribute to the modulation of the joint inflammatory response. There is considerable evidence to support the clinical efficacy of the two components when used separately or in association to other supplements in dogs with osteoarthritis [21–30], but no study has been carried out looking at the combined effect of the two components. To the authors’ knowledge, this is the first study to test the effects of a feed supplement containing undenatured Type II Collagen (UC II^®^) and *Boswellia Serrata* for managing mild to moderate mobility disorders in dogs.

The purpose of this study was to evaluate the effects of this compound on mobility, pain and joint metabolism in canine osteoarthritis (OA). The hypothesis was that a combination of UC-II^®^ and *Boswellia serrata* would be more efficient in enhancing mobility, pain relief, and joint metabolism among dogs affected by mild/moderate OA as compared to a placebo. To test this hypothesis owner and veterinarian´s evaluations together with objective tools were used. Particularly, mobility was assessed by Liverpool osteoarthritis in dogs questionnaire (LOAD score), accelerometry (Actical Activity, AC) and baropodometric gait analysis (Gait Lameness score—GLS); pain was assessed by the clinical evaluation, Von-Frey filament test and number of OA crisis recorded; the metabolic profile of the joint was assessed by metabolomic analysis of the synovial fluid of the most affected joint.

## Material and methods

The study was designed as a single-center, randomized, double-blind, placebo controlled, cross-over clinical study, and approved by the ethics committee (approval n. 03.2019). Recruitment began in September 2020 and ended in October 2021. For each dog, the owner received a detailed written description of the study protocol and gave written informed consent prior to enrollment in the study. Dogs could be withdrawn from the study at any time at the owner’s request. If a dog was believed to need additional pain management while included in the study, except rescue treatment (see below) or severe adverse effects occurred, unblinding would occur, and the dog would be excluded and treated with standard-of-care analgesics.

### Population of the study

For the purpose of the study, client-owned dogs aged > 1 year, weighing ≥ 5 kg, with a medical history, clinical signs, mobility impairment attributable to OA and referred to the Surgical Unit of the Section of Veterinary Clinics of the Department of Emergency and Organ Transplantation of the University of Bari were screened. Only dogs with presence of clinical and radiographic signs of osteoarthritis, with a COAST score 2 or 3 (mild/moderate OA) were considered for eligibility [9]. The exclusion criteria were absence of signs of osteoarthritis, presence of inflammatory arthropathies nonrelated to OA, COAST score of 4 (severe OA), recent history of joint traumatism, presence of intraarticular treatments in the last year, presence of a cardiovascular disease clinically important (higher than B2 based on the ACVIM consensus score), severe dental disease, presence of neurologic, renal, hepatic and chronic respiratory disease. During the screening visit, each dog underwent a full clinical, orthopedic and x-rays evaluation. In addition, haematological and biochemical examinations were performed to assess the health status of the dogs and the meeting of the inclusion criteria.

### Study protocol

At time of the inclusion, a progressive ID number was assigned to each dog. Randomization was performed by the sponsor in order to keep the product blind for the owner and also for the investigator. Dogs were randomized in a ratio 1:1 to receive product A (UC II^®^ + *Boswellia* supplement) or product B (placebo), 1 chew per day, for 8 weeks by oral route. The supplementation was repeated in a crossover design after 4 weeks washout period. After the first evaluation (Baseline, T0) dogs were reassessed every 7 days by phone interview and every 4 weeks (T1, T2, T3, T4 and T5) with a complete examination, for the evaluation of the effect of the therapy and the evolution of the disease, for a total period of 20 weeks. A visit window of +/-5 days was accepted for all the visits. Any delay in scheduled visits, collateral or concomitant effects, analgesia rescue administration and other issues were immediately communicated to the study manager (within 24 hours), and if required, the pharmacy- surveillance system was alerted.

### Oral supplement

The formulation of the commercial product (Flexadin Advanced *®* Boswellia**)** consisted in an association of: Omega 3 fatty acids total (84 mg), *Boswellia Serrata* (190 mg), undenatured Collagen type II (UC-II®) (40 mg), eicosapentanoic acid (EPA) (9,3 mg), Vitamine E (22,5 UI), Chicken flavour q.s.p., for each 3 g chew. The placebo chew did not include any active compounds.

The chews were provided in such a way that the owner was not able to understand which type of supplement the dog was receiving. At each scheduled visit, owners brought the bags with remaining product, allowing the dispenser to verify that the dog had received the product assigned.

### Clinical assessment

#### Owner assessment

At each clinical evaluation, the owners were asked to complete the Liverpool Osteoarthritis in Dogs (LOAD) questionnaire. The LOAD [11] consists of 13 items, all of which are scored on a five-point scale. The score range per item is 0 to 4, and the summed item scores yield the total score for the instrument. Based on the overall score each patient was assigned to a specific grade of alteration of the mobility: not affected (0), mild (< 11), moderate (11–20), severe (21–30), extreme (31–52). The questionnaire was also proposed to the owners weekly by a phone interview. A discomfort grading was also performed, asking to the owners to give a grade of severity from 1 to 4 according to the animal’s status. The LOAD score obtained at 4 and 8 weeks of each supplementation period was considered, and the percentage variation compared to the respective baseline was computed.

#### Veterinarian assessment

All dogs underwent a complete physical examination at each scheduled visit. The orthopedic examination included the evaluations of posture, mobility, pain at the manipulation of the affected joint and assessment of the range of motion (ROM). The mobility evaluation was performed, at the walk and trot, to classify dogs’ lameness as following: no visible lameness; temporary (temporary mild/moderate lameness triggered by the physical exercise); persistent (persistent mild/moderate lameness with episode of acute severe lameness); severe and persistent (severe and persistent lameness and/or with the involvement of more than one joint). These grades of severity were scored from 1 to 4.

In addition, the suspected osteoarthritic joint(s) were evaluated with a radiographic examination, and when OA diagnosis was confirmed, the x-ray was graded based on the following scheme: 1 = no signs; 2 = mild: evidence of articular incongruence, possible subchondral sclerosis, absence of osteophytes; 3 = moderate: evidence of articular incongruence, evident subchondral sclerosis, scarce osteophytes; 4 = severe: evidence of articular incongruence, evidence of subchondral sclerosis, presence of osteophytes, bone deformations.

All assessments were carried out by the same clinician with specific and longstanding expertise in the management of OA.

Based on the data collected during the first physical and radiographic examinations the degree of the OA was scored considering the Canine Osteoarthritis Staging Tool (COAST): pre-clinical (stage 0–1), At risk (stage 1), mild (stage 2), moderate (stage 3), severe (stage 4), considering only the stage 2 and 3 for the purpose of the study [9,31].

#### OA crisis assessment

In case of an increase of the LOAD score more than 20% of the baseline value, based on the visit or phone interview, Paracetamol 10 mg/kg BID was administered for three days. These episodes were considered as OA crisis and their total number of OA crisis were considered as an endpoint, in order to assess the efficacy of the supplementation.

### Instrumental assessment

#### Mobility assessment (GAIT4 Dog^®^ analysis)

A commercially available pressure mat walkway, the GAIT4 Dog R walkway (CIR Systems Inc.,Sparta,NJ), consisting of a 5.8 × 0.6m portable mat with 18,432 encapsulated sensors, connected to a dedicated software (GAITFour^®^ software version 4.9Wr, CIR Systems Inc., Sparta, NJ, United States), was used to record and evaluate spatial, temporal, and pressure variables for gait analysis [32]. The mat was calibrated by the manufacturer before use. The test was performed at every scheduled visit (T0; T1, T2, T3, T4, T5). Before to perform it, each dog was familiarized with the mat for at least 10 minutes, and then walked on the mat at least 10 times. When possible, the test was performed without a leash, to avoid any external influence. Recorded walks were processed by the dedicate software and then accurately evaluated by an expert clinician. Video recordings were reviewed to ensure the right readings. Walks in which the dog exhibited altered behaviors such as stopping, trotting, pulling on leash, turning head significantly, having inconsistent velocity, or less of three gait cycles were excluded according to the manufacturer’s recommendations. For each dog and each time, the analysis was based on an average of five valid and representative walks, consisting of at least three gait cycles, correctly processed, with an internal velocity of less than 10%. All tests were supported by a video recording, captured using a fixed camera placed at a height of 50 centimeters from the floor. GAIT4 Dog^®^ Lameness Score (GLS), was calculated by considering weight distribution, based on observed-to-expected total pressure index (TPI) by limb and established body type loading ratios (default 60:40) and should be approximately 100%, considering the absence of lameness values above 95%.

#### Activity assessment

An accelerometer (Animal-Actical^®^, Activity Monitor Device, Respironic, Bend, Oregon) was utilized to assess the activity levels of dogs, constantly measuring the intensity, frequency, and duration of movement. The system works by using a piezoelectric sensor that generates a voltage when the device is subjected to a change in speed per unit of time, allowing it to process movement in all directions. The voltage is then digitized. For each epoch, a one-minute measurement period was carried out. The digital value during this period was compared to the baseline value, and the difference between them was utilized to formulate a raw activity value for the particular interval. Subsequently, the raw activity value was transformed into an activity count by employing standard software.

The activity monitor (AM) was placed on the collar and positioned ventrally on the neck of 8 of the study dogs after enrolment and 1 week before the start of supplementation to record baseline activity. The dogs constantly wore the AM throughout all phases of the study, and it was removed only at each visit to download the record, reset the memory, and check the battery level. For the analysis the day records were divided in three windows Q1 (0–8), Q2(8–16), Q3(16–24). To assess the ability of the AM to detect an overall effect of supplementation, the change in activity count (AC), for each time window was compared between groups.

#### Pain assessment

An electronic Von Frey (EVF) (eVF Electronic Von Frey, Ugo Basile, Italy) was used in this study to assess the pain threshold of the most affected joint.

The device consists of a weight cell, a 0.8 mm diameter rigid nitinol (metal) stimulation filament, a prism and a pedal for manual scoring.

The test was performed in a quiet room with the animal unrestrained in a standing or lateral position. The probe’s maximum force was set to 1000 g with a sensitivity of 50 g. The examiner administered an increasing force (ranging between 0.1 and 1000 g) to the selected joint’s lateral skin, until they elicited a withdrawal reflex or an equivalent painful reaction within a 25-second time limit. Moreover, any behavioral response indicating a conscious pain perception was marked as the endpoint of the stimulus, such as limb movement away from the probe, turning the head to look directly at the site of stimulation, vocalization, etcetera. The greatest force applied to the limb was recorded digitally using an internal load cell connected to the tip and the recording device. To save force curves, peaks and export data to Excel files, a specific software (Ugo Basile, Italy) was used.

In addition to the selected joint, the test was conducted on the other two joints (proximal and/or distal) of the same limb. Three measurements of the same area were taken at each visit and a mean value was calculated for the analysis.

#### Metabolomic assessment

To investigate the metabolomic profile by ^1^H-NMR analysis, all dogs included in the study underwent synovial fluid (SF) sampling. The sampling was performed at the beginning (T0) and at the end of the study (T5), considering the most affected joint. Arthrocentesis was performed after X-ray examination, with patients heavily sedated or under general anesthesia. The joint to be sampled was clipped and aseptically prepared. The procedure was always performed by the same operator (LC). For sampling, needles of appropriate length were chosen related to the size of the patient and the anatomy of the interested joint. Each sample collected was stored in a sterile Eppendorf tube at a temperature of − 20°C until the ^1^H-NMR measurements. Samples were spun down in a micro centrifuge at 14000 rpm for 15 minutes (temperature 4°C). Then, 420 μL of each synovial fluid sample were added of 280μL D_2_O containing TSP as a chemical shift reference, d = 0 ppm, and filled in a 5 mm NMR tube. The analysis of SF samples was performed at the General and Inorganic Chemistry Laboratory of Department of Biological and Environmental Sciences and Technologies (DiSTeBA), University of Salento.

For each sample a 1D ^1^H one-dimensional CPMG experiment with a transverse-relaxation-filter incorporating pulse sequence (referred to as Carr–Purcell–Meiboom–Gill spin-echo sequence, CMPG) was run with 64 scans, 64 K time domain, spectral width 20.0276 ppm (12,019.230 Hz), 2 s delay, p1 8 μs and 2.73 s acquisition time. The resulting FIDs were multiplied by an exponential weighting function corresponding to a line broadening of 0.3 Hz before Fourier transformation, automated phasing, and baseline correction. Metabolite identifications were based on ^1^H and ^13^C assignment by 1D and 2D omo and heteronuclear experiments (2D ^1^H Jres, ^1^H COSY, ^1^H-^13^C HSQC, and HMBC) and by comparison with the literature data [33].

Spectra acquisition was performed at 300 K on a Bruker Avance III 600 Ascend NMR spectrometer (Bruker, Ettlingen, Germany), operating at 600.13 MHz for 1H observation, equipped with a TCI cryoprobe incorporating a z axis gradient coil and automatic tuning matching (ATM).All the spectra were recorded at 300 K on a Bruker Avance III NMR spectrometer (Bruker, Karlsruhe, Germany), operating at 600.13 MHz for^1^H observation, equipped with a TCI cryoprobe incorporating a z axis gradient coil and automatic tuning-matching (ATM).

### Statistical analysis

Data analysis was performed using MedCalc software (Vers. N. 14).

A two-crossover sample means approach was used to calculate the sample size with an alpha of 0.05 and a power of 0.8. A total of 13 cases has been calculated. Considering a 20% drop-off rate a total of 16 cases should be recruited for the study. Shapiro-wilk test was performed for each variable to test normality. When the normality assumption was verified pairwise t-test was performed to find the difference before and after supplementation. For non-normally distributed data non-parametric Kruskall-Wallis test was performed. A mixed model for a repeated-measures ANOVA, followed by Bonferroni post-hoc adjustment, was used to analyze differences among the groups of the study, considering the effects of treatment and time. Values of *p* ≤ 0.05 were considered significant. The ^1^H NMR spectra were processed using Topspin 3.6.3 and Amix 3.9.13 (Bruker, Biospin, Italy). The entire NMR spectra (in the range 10.0–0.9 ppm) were segmented in fixed rectangular buckets of 0.04 ppm width. The total sum normalization was applied to minimize small differences due to sample concentration and/or experimental conditions among samples. The data set (bucket table) resulted in a matrix, corresponding to the bucketed ^1^H NMR spectra values (in columns), measured for each sample (in rows). Multivariate statistical analysis (unsupervised principal component analysis (PCA) and the supervised orthogonal partial least squares discriminant analysis OPLS-DA) was performed to examine the intrinsic variation in the data, using SIMCA 14 software, (Sartorius Stedim Biotech, Umeå, Sweden). The Pareto scaling procedure was applied, performed by dividing the mean-centered data by the square root of the standard deviation. The robustness of the statistical models was tested by cross-validation default method (7-fold) and further evaluated with permutation test (400 permutations). The quality of the models (the total variations in the data and the internal cross validation) was described by R2 and Q2 parameters. The relative change in discriminating metabolites between the observed groups was evaluated by analyzing the mean values +/- standard deviation of selected bucket reduced distinctive unbiased NMR signals after spectra normalization (to the total spectrum excluding the residual water region) [34]. In particular, the changes in metabolite levels between two groups were calculated as Log2 fold change (FC) ratio of the normalized median intensity of the corresponding signals in the spectra of the groups [33,35,36]. Statistical significance was set at least at an adjusted p-values < 0.05.

## Results

**Fig 1:**
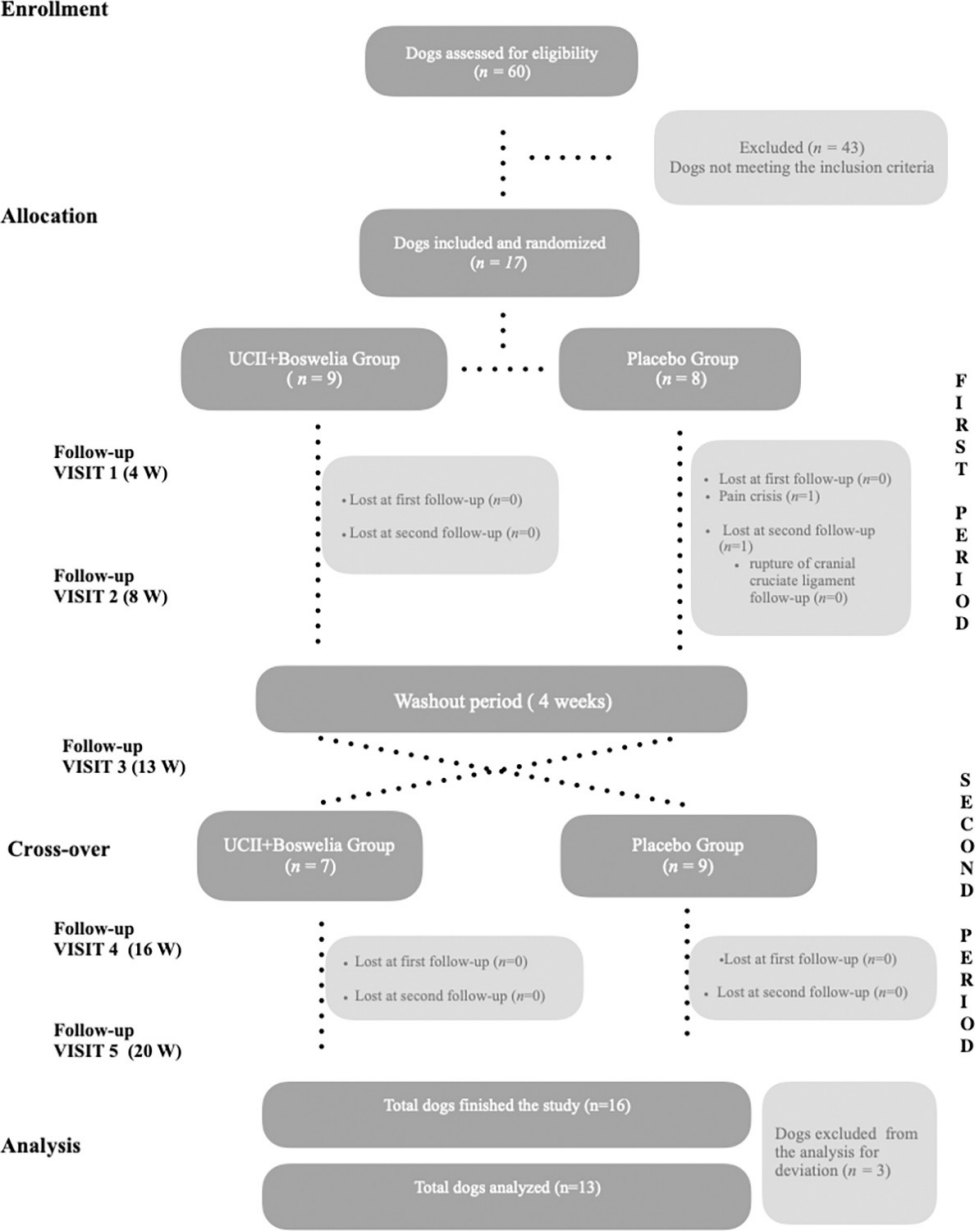
Flow chart of the study, performed following the CONSORT guidelines [37].

A total of 60 dogs with mobility problems were evaluated and enrolled in the study. After the first screening, only 17 of these dogs met the inclusion criteria. They were then included in the study and randomly assigned to different treatment groups, as illustrated in Fig 1. In the first period 9 dogs received UCII^®^+ *Boswellia serrata* supplement (product A) and 8 dogs the placebo (product B), while in the second period 7 dogs received product A and 9 dogs product B. During the first period of supplementation, one dog (Patient ID 10) of placebo group was lost at the second follow-up visit (T2) for a worsening in lameness condition due to a traumatic rupture of cranial cruciate ligament.

All owners reported good palatability and ease of administration of the tested dietary supplement. No adverse effects (vomiting, diarrhoea, anorexia), monitored daily by the owners, were observed.

A total of 16 dogs completed the study, and their data were analysed. After a preliminary analysis of the data, the authors excluded three dogs from the study as they had some deviations recorded during the study, such as more than one late visit (patient IDs 9, 11, 17), which could affect their data. A final analysis was then conducted on data from 13 dogs. The population of this study consisted of 5 females (1 intact and 4 sterilised) and 8 males (6 intact and 2 neutered) dogs with a mean age of 6.3 ± 4.1 years, weighing 30.5 ± 10.4 kg and with a BCS of 5.2 ±1.1, suffering by mild (Coast 2. n = 1) and moderate (Coast = 3, n = 12) osteoarthritis. Among the entire population the most affected joint was the hip (n = 11) followed by the elbow (n = 2). Xray alterations were scored as mild (n = 1) and moderate (n = 12) (Table 1).

**Table 1:** Demographic characteristics of the study dogs. Data for age, weight and body condition score (BCS) are expressed as mean and standard deviation.

Dogs	Included (n = 17)	Analyzed (n = 13)
**Age (years)**	6 ± 3.9	6.3 ± 4.1
**Sex (M/F)**	10/7	8/5
**Weight (Kg)**	33 ± 10.7	30.5 ± 10.4
**BCS (/9)**	5.8 +1.1	5.2 ±1.1
**Breed (n)**	Mix breed (4) Labrador (2) Rottweiler (2) German Shepard (2) English Setter (2) Border Collie (1) Bernese Mountain Dog (1) Italian Mastiff (1) Cocker Spaniel (1) Siberian Husky (1)	Mix breed (4) Labrador (2) German Shepard (1) English Setter (2) Border Collie (1) Bernese Mountain Dog (1) Italian Mastiff (1) Cocker Spaniel (1)
**Joint affected (n)**	Hip (13) Elbow (2) Knee (2)	Hip (11) Elbow (2)
**COAST stage (n)**	COAST 2 (2) COAST 3 (15)	COAST 2 (1) COAST 3 (12)

**Fig 2:**
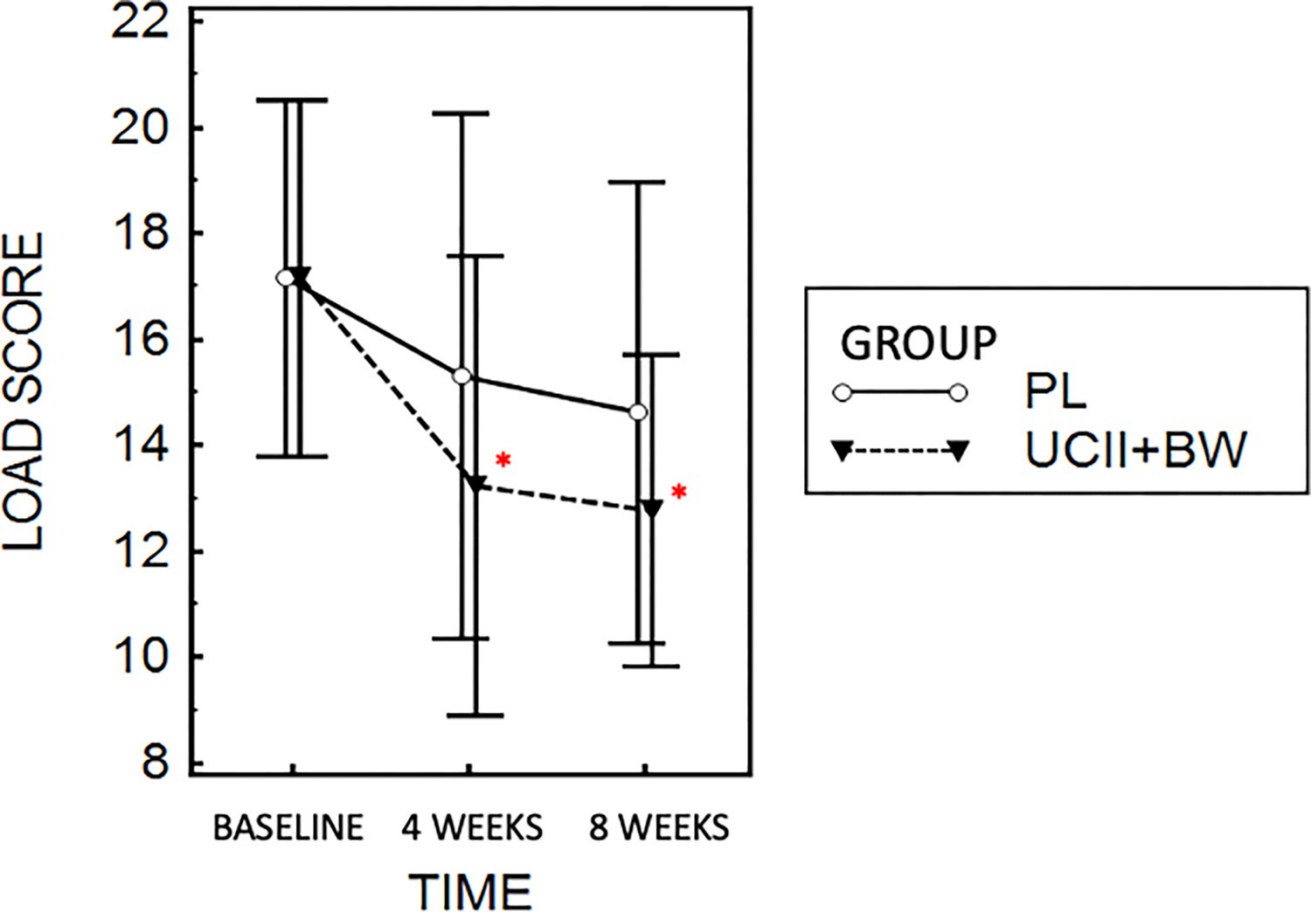
LOAD score variation during UC-II^®^- Boswellia Serrata and placebo administration. Data are presented as mean and 95% of confidential interval. * *p* < 0.05 compared to baseline.

**Fig 3:**
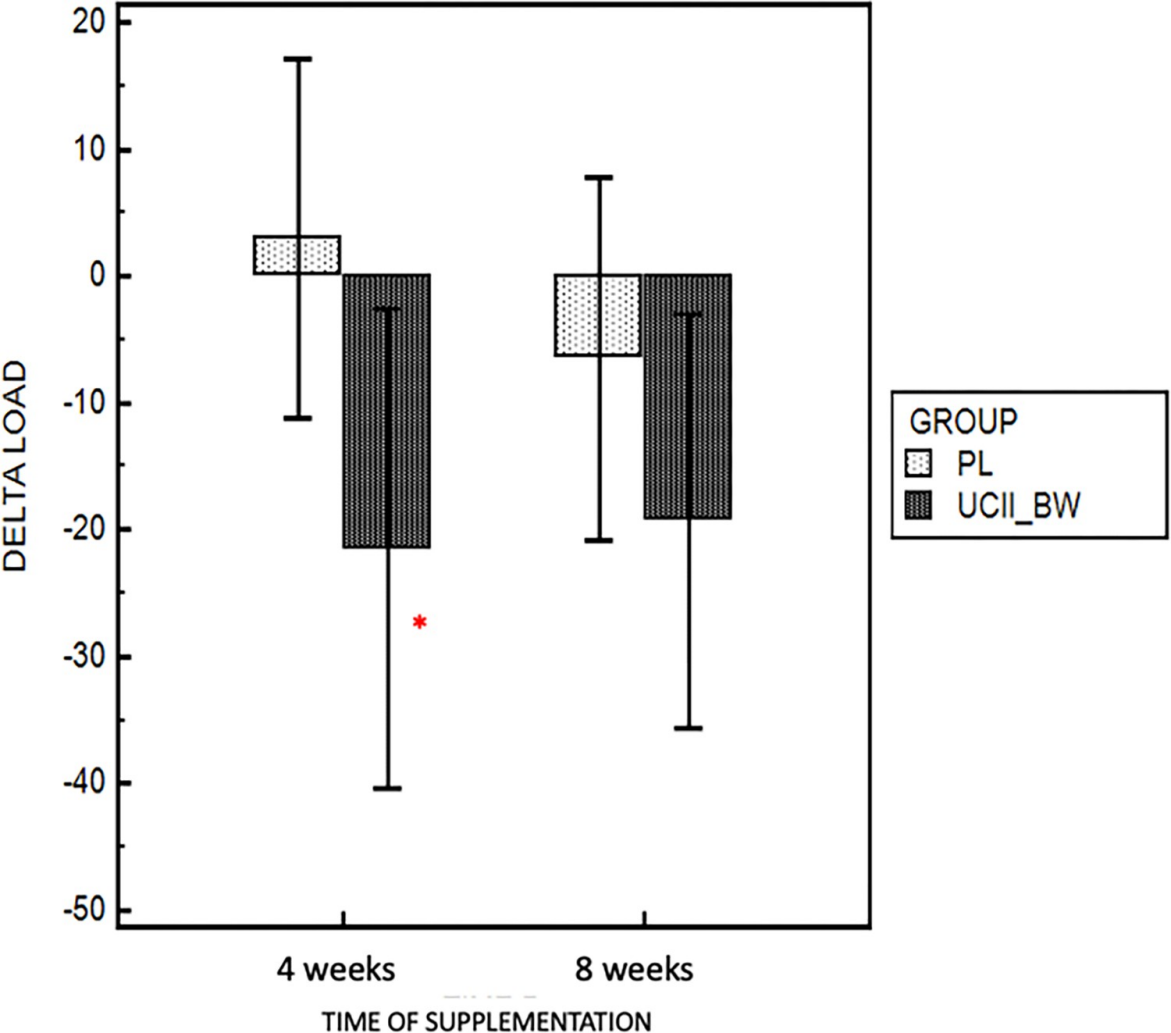
Percentage variation of LOAD score at 4 and 8 weeks of supplementation in UCII^®-^ BW and placebo group. * *p* < 0.05 compared to placebo.

Results based on LOAD assessment showed a significant reduction in absolute LOAD values during supplementation (UCII®-BW group) at 4 (p = 0.02) and 8 weeks (p = 0.01) compared to baseline (T0) (Fig 2). No significative differences were observed in the placebo group between baseline and the washout period (p = 0.38). Looking at the score in terms of percentage variation compared to the respective baseline assessment, the UCII®-BW group showed a greater reduction in delta score at 4 (- 21.4%, p = 0.03) and 8 (- 19.3%, p = 0.21) weeks compared to the placebo group (+ 2.9% and -6.5%) (Fig 3).

Considering the veterinarian assessment, data of posture, mobility, pain and ROM evaluation at different times and groups are showed in Table 2.

**Table 2:** Data of different outcomes are reported as median and 95% CI for LOAD, posture, mobility, pain and ROM and mean and 95% CI for GLS, AC and Von Frey grams, at baseline and during different groups and times of administration.

	Baseline	UCII^®^-BW (4week)	UCII^®^-BW (8week)	Washout	Placebo (4weeks)	Placebo (8week)
**Owner assessment**						
**Load**	17 (12–21)	11 (8–17)	13 (8–15)	14 (10–19)	15 (9–19)	15 (8–21)
**Veterinarian assessment**						
**Posture**	2 (2–2)	2 (2–2)	2 (2–2)	2 (1.26–2	2 (2–2)	2 (2–2)
**Mobility**	2 (2–3)	2 (2–2.5)	2 (2–2)	2 (2–2.5)	2 (2–2.5)	2 (2–2.5)
**Pain**	2 (2–3)	2 (2–3)	2 (2–2.4)	3 (3–1)	2 (1.75–3)	3 (2–3.4)
**ROM**	3 (2–3)	2 (2–3)	2 (2–3)	2 (2–3)	3 (2–3)	3 (2–3)
**Instrumental assessment**						
**GLS**	93 (90–97)	96 (92–105)	99 (89–102)	92 (87–101)	95 (89–101)	98 (90–102)
AC Q1 Q2 Q3	6120 (4900–7340) 6178 (5022–7333) 6185 (4898–7472)	6645 (4410–8880) 6975 (4603–9347) 6916 (4677–9155)	5963 (3426–8500) 6077 (3460–8696) 5991 (3413–8569)	7477 (4657–10297) 7570 (4880–10260) 7570 (4870–10268)	7657 (2486–12829) 7740 (2685–12794) 7766 (2746–12786)	7058 (2972–11143) 7117 (3132–11102) 7193 (3275–11112)
Von Frey (g)	355 (275–436)	317 (234–400)	393 (293–493)	311 (251–372)	212 (153–271)	327 (227–428)

No differences were found between times and groups of supplementations (p = 0.38).

An interesting trend was recorded for pain and ROM score, that showed higher values during placebo administration and washout period, even if not statistically significant when compared between groups.

One case of an OA crisis in a dog (ID13) needed the use of a rescue analgesic (Paracetamol 10 mg/kg BID for 3 days). The analysis revealed that it occurred while the dog was taking the placebo supplement. Other animal subjects did not experience any crises linked to OA pain.

**Fig 4:**
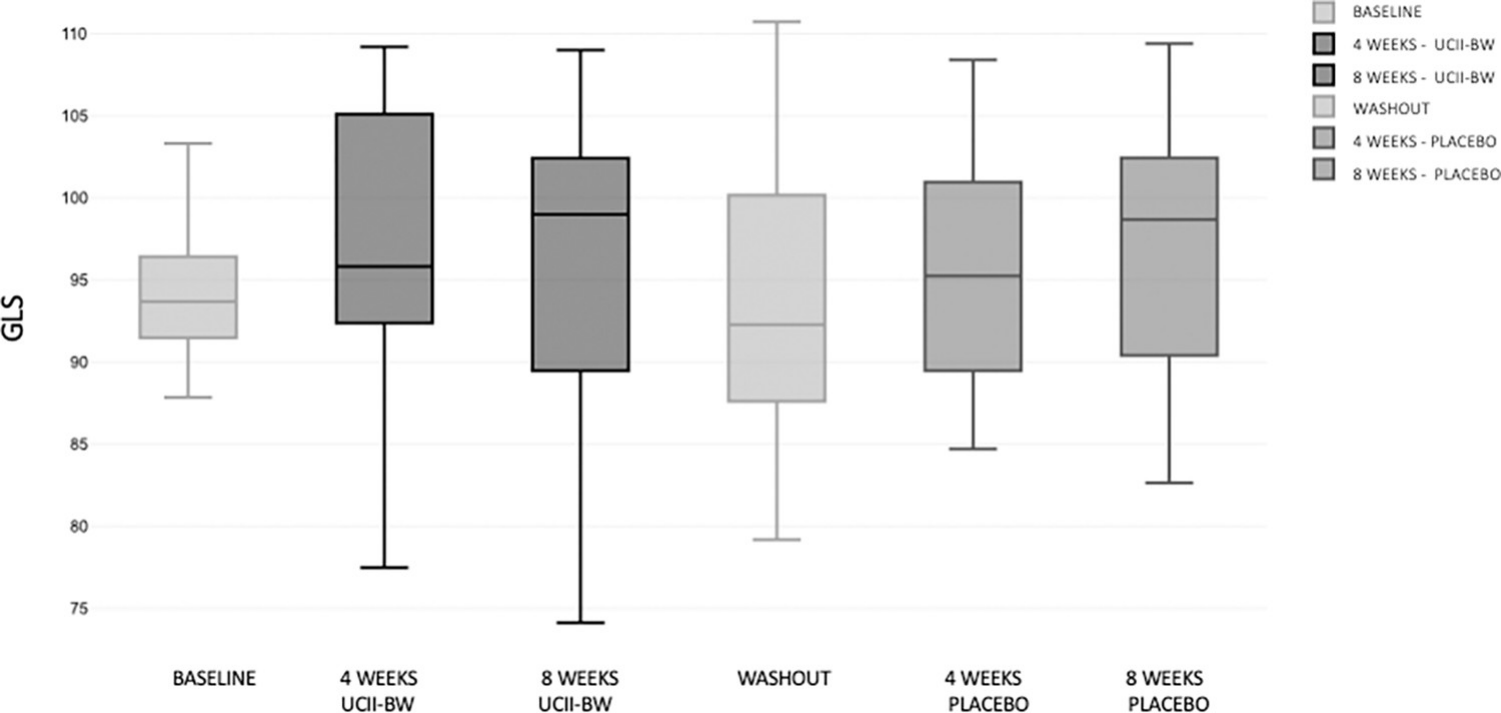
GLS variation during different times of administration and washout period.

For the gait analysis, TPI was excluded from the analysis due to the different localization of the disease in our population. For this reason, GLS was considered as parameter that better reflected the dynamic weight bearing, equally for each limb. However, there were no significant differences between time and groups in GLS score (Fig 4).

The results of mobility activity reveal no difference between groups or time of supplementation, considering the same hourly windows (Q1 (00:00–07:59), Q2 (8:00–15:59), and Q3 (16:00–23:59). Due to technical issues, three activity monitors were excluded, and data was lost. Thus, the final analysis involved the examination of records from five devices.

**Fig 5:**
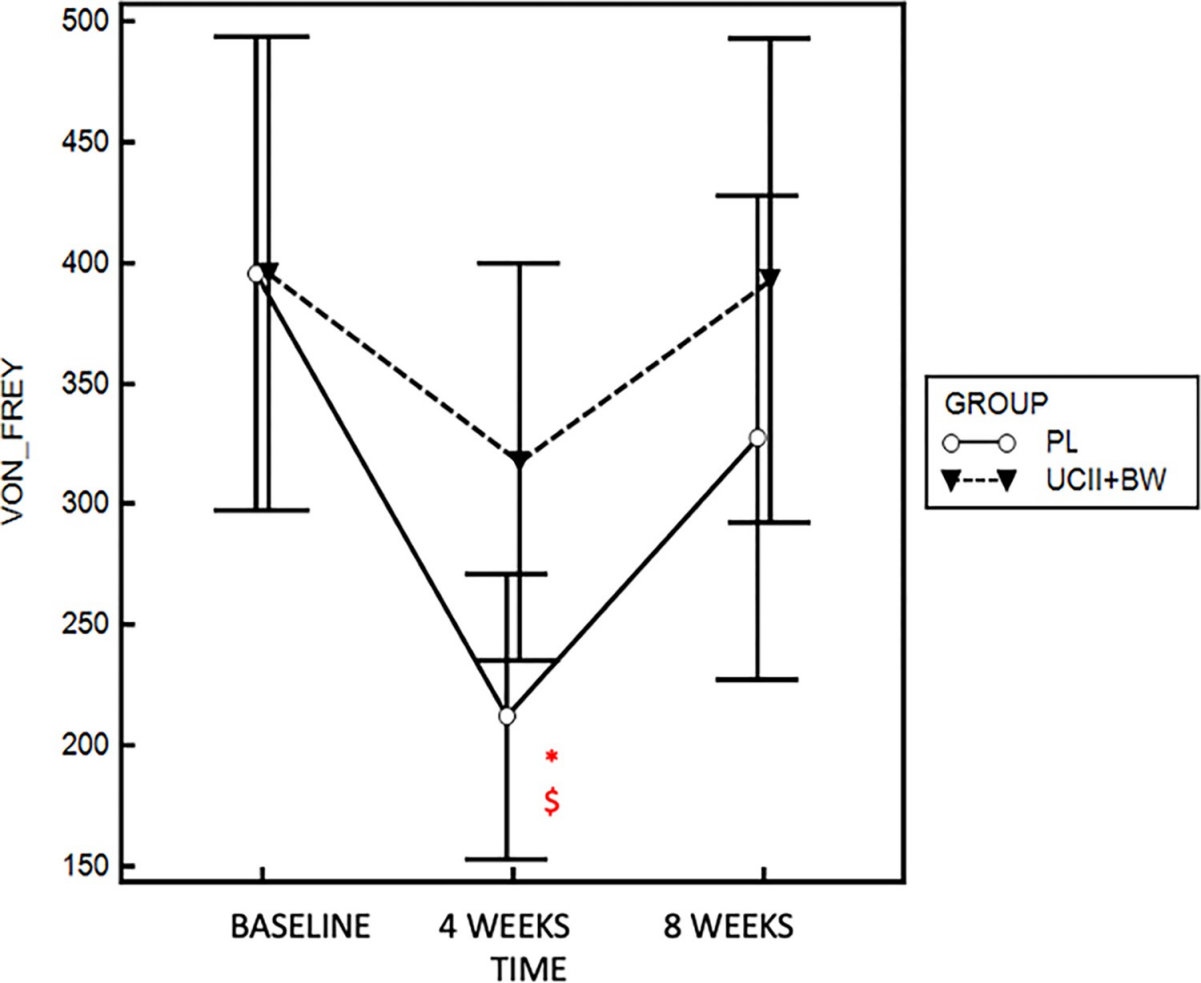
Von Frey grams variation during UCII^®^-BW and placebo administration. * *p* < 0.05 compared to baseline. $ *p* < 0.05 compared to placebo.

The mean sensory pain threshold values of the most affected joint showed a particular trend in both groups, with a first reduction recorded after 4 week of supplementation and a restoring of original condition after 8 weeks. Values in the placebo group were found significative lower after 4 weeks of administration when compared to UCII^®^-BW group (317 ± 123 g, *p* = 0.04 and to baseline (356 ± 120 g, p = 0.003) (Fig 5).

Metabolomics data were obtained from a total of 24 synovial fluids (SFs), with 14 collected at baseline and 10 at the end of the study. Due to the joint condition, it was not possible to obtain SFs from each dog at every time point. Complete sampling was obtained for 9 patients.

Due to a high content of water (H_2_O) or blood, certain samples (9 SFs collected at T0 and 2 at T5) could not be distinguished spectrally and were therefore excluded from the analysis.

**Fig 6:**
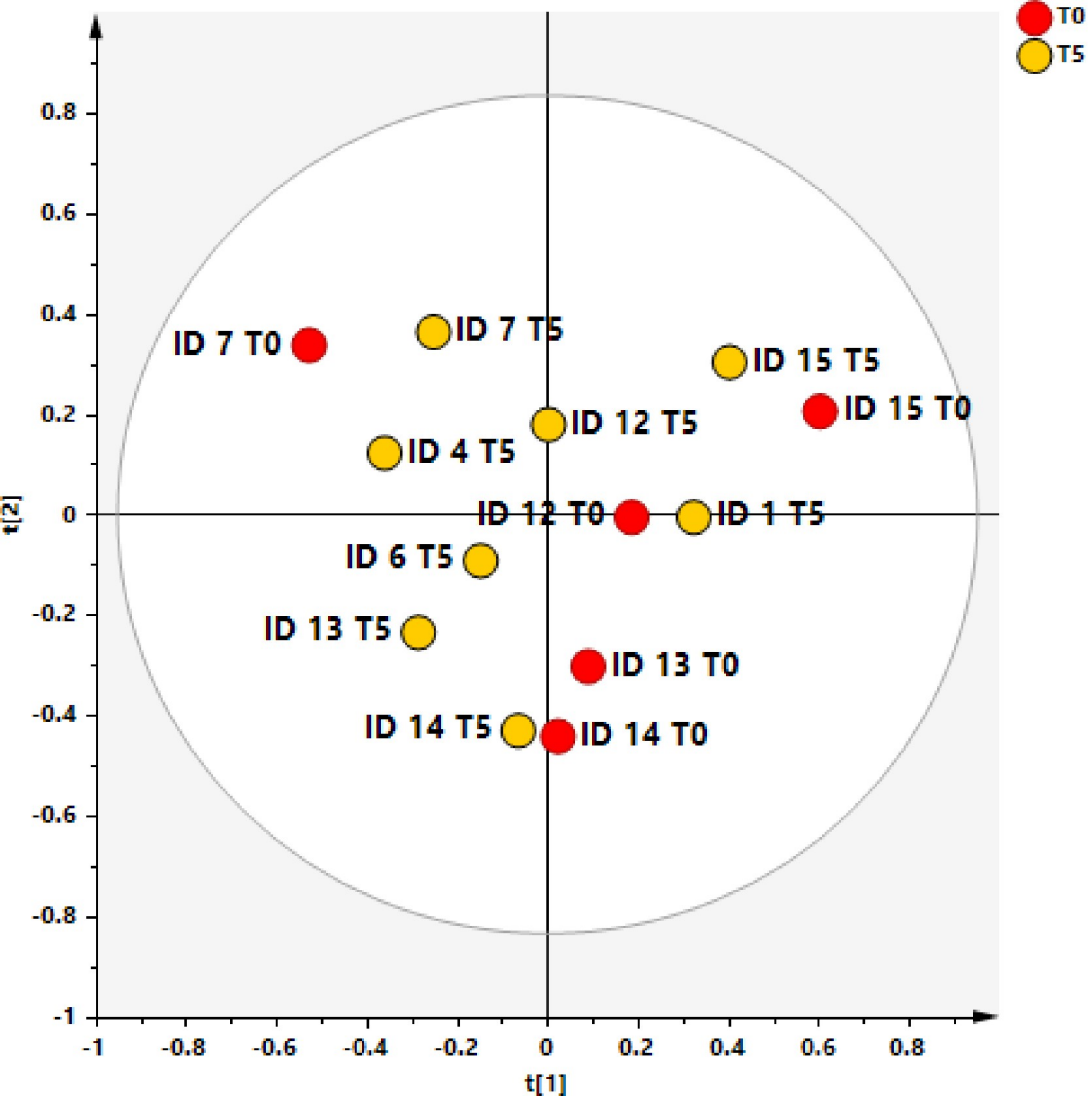
PCA scores plot for the whole data set. Three components give R2X = 0.739 Q2 = 0.16. Symbols are colored according to sampling time: T0 (baseline–pre supplementation) and at the end of the study (T5).

**Fig 7:**
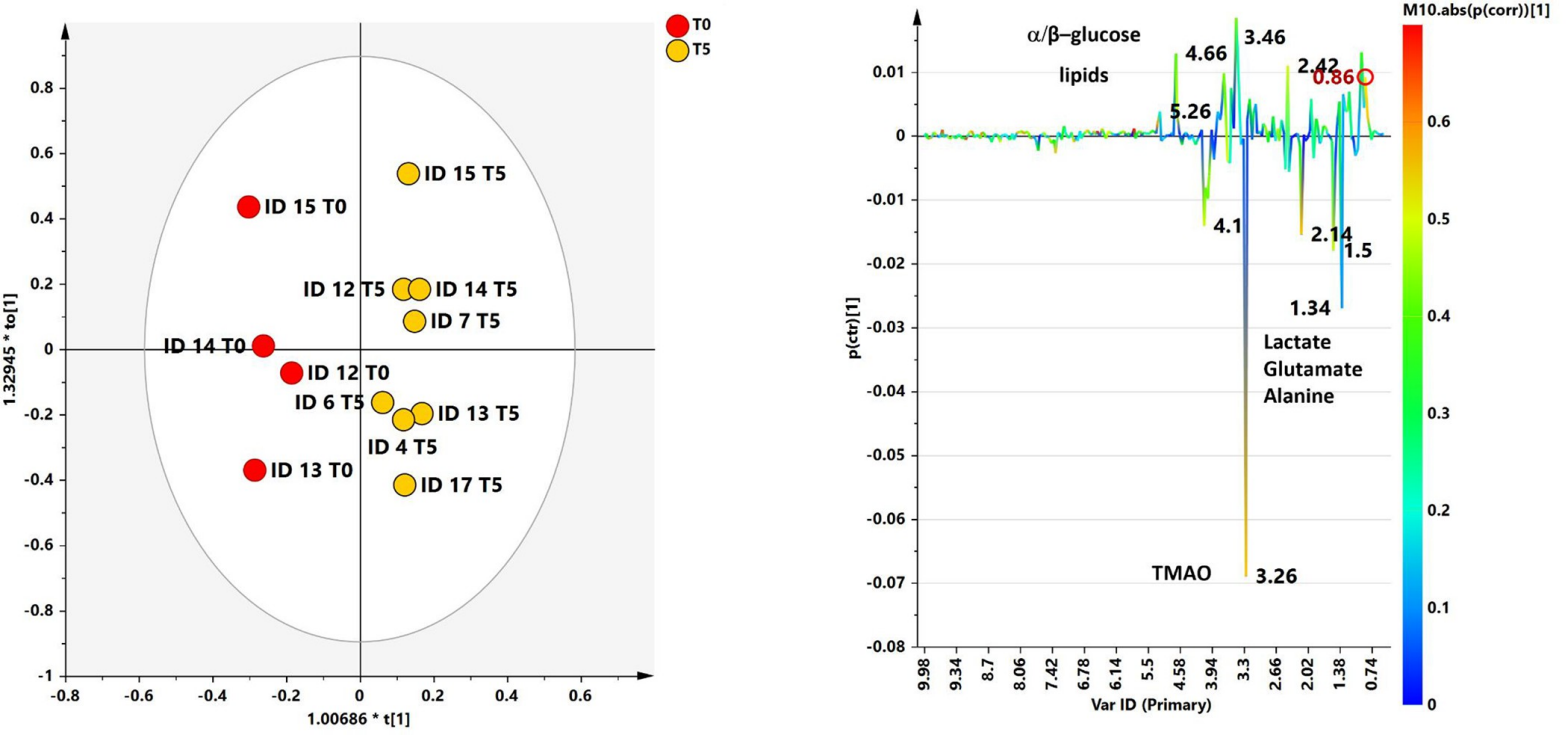
OPLS-DA t[1]/t[2] scores plot for T0 and T5 SF class samples. (b) S line plot for the model colored according to the correlation-scaled coefficient. The color bar indicates the correlation of the metabolites discriminating among classes.

The ability of ^1^H‐NMR‐based multivariate analysis to discriminate SF samples from different sampling time (T0 and T5) and randomized dogs, receiving UC-II^®^-BW supplement or placebo (AB or BA), was used by performing principal component analysis (PCA) (Fig 6) followed by orthogonal partial least square discriminant analyses (OPLS. DA) (Fig 7).

The preliminary unsupervised PCA analysis gave a scores plot (R2X(cum) and Q2(cum) of 0.739 and 0.160, respectively) exhibiting an expected clustering of the samples (T0 and T5) for each patient. Anyhow, the same model suggested, except for ID7 T0 sample, a specific separation between T0, pre and T5 (post) SF samples (Fig 7).

**Fig 8:**
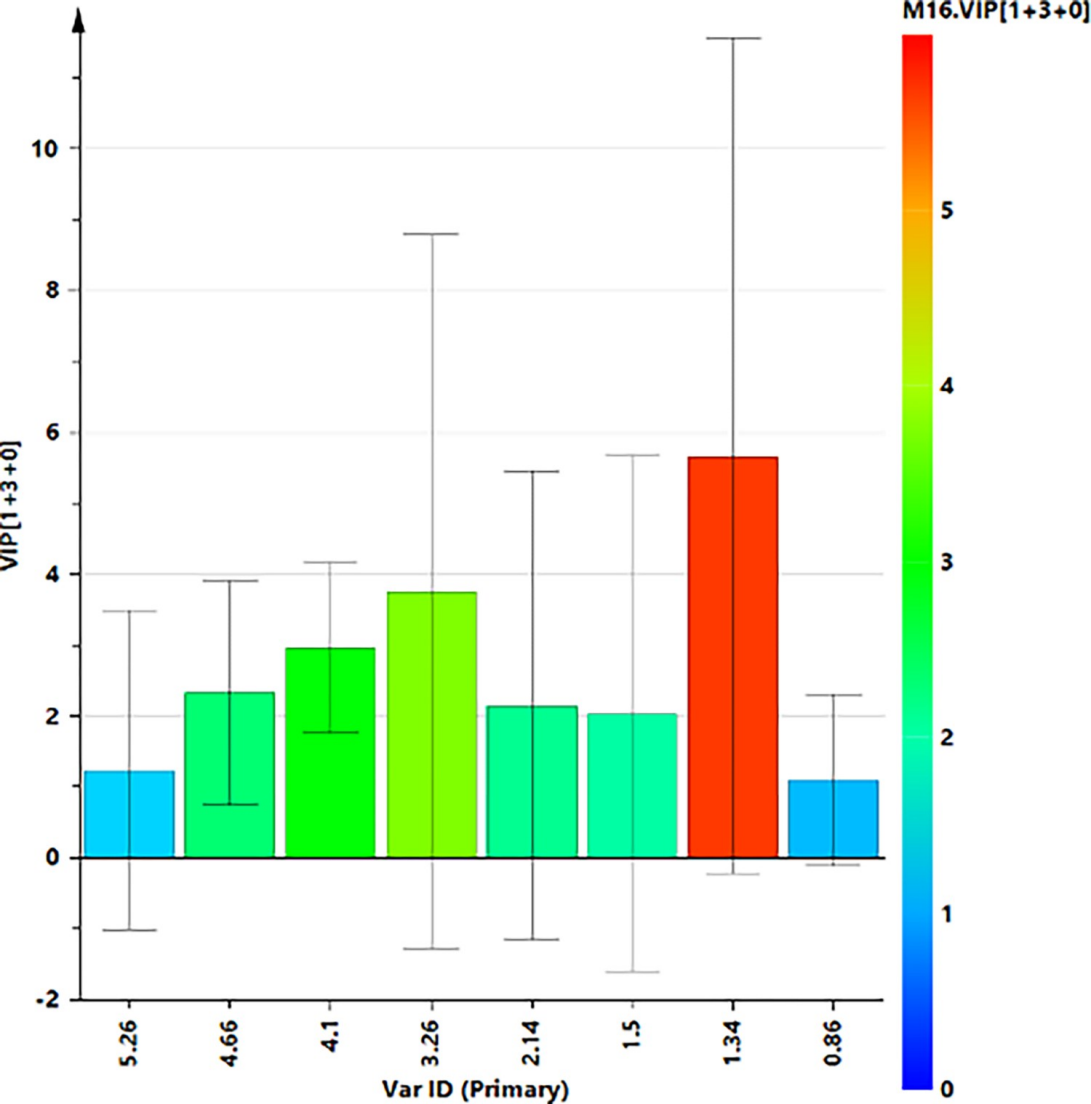
VIP plot displays the VIP values (≥1) as a column plot with jack-knife uncertainty bars (95%).

**Fig 9:**
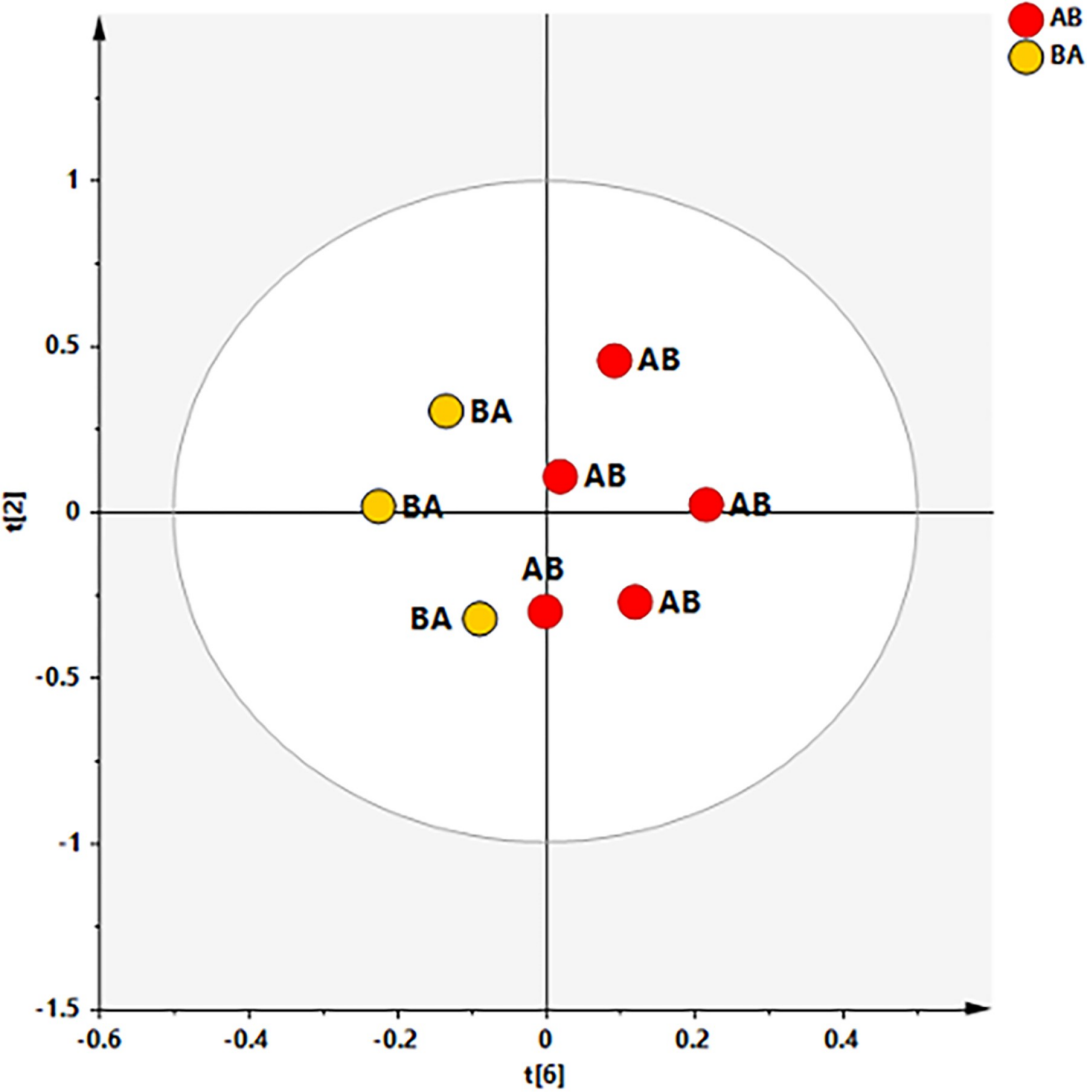
PCA scores plot for the whole data set. Six components give 6 components give R2X = 0.962 Q2 = 0.398. Symbols are colored according to placebo/treatment administration time: AB (treatment, placebo) and BA (placebo, treatment).

A supervised OPLS‐DA (number of latent variables 1 + 3 + 0) was performed with the aim to enhance the separation between T0 and T5 SF samples. The resulting OPLS‐DA score plots showed a discrimination between SF group samples with moderately satisfactory predictability (Q2 = 0.16) and goodness of fit values (R2X and R2Y of 0.68 and 0.96, respectively). The corresponding S line plot of the loading vectors for the first components, colored according to the pcorr values, showed the molecular components responsible for the observed separation among the samples. Potential differential compounds were selected with strong discrimination power (VIP ≥ 1) (Fig 8), identifying a total of seven discriminating metabolites (Fig 9 and Table 3). Particularly, lactate, glutamate, alanine and TMAO, were found to be higher in baseline time samples (T0), while α and β glucose, lipids, were higher for T5 samples.

**Table 3:** Fold change calculated as the ratios between the two group means (T5/T0). The significant features are beyond the given threshold (FC = 1).

Metabolites	Chemical shift (ppm)	Fold change ratio (T5/T0)	LOG2 FC
**lipids**	0.86	1.37	0.46
**alanine**	1.5	0.45	-1.16
**glutamine**	2.14	0.58	-0.78
**TMAO**	3.26	0.82	-0.29
**lactate**	4.1	0.61	-0.71
**a glucose**	4.66	1.31	0.38
**b glucose**	5.26	1.59	0.67

Discriminating constituents between T0 and T5 classes were then quantitatively compared by considering the fold change (FC) ratio. Among selected seven metabolites with strong discrimination power only lactate (4.1 ppm) difference reach statistical significance (p ≤ 0.05) (Table 3).

Considering the choice of sampling just at the beginning and at the end of the study (and not before and after each 8 weeks of supplementation), we also investigated the possible metabolic change related to the time of the effective UC-II^®^-BW supplementation following the scheme AB or BA, (see experimental procedure). Based on the unsupervised analyses, performed by considering T0 (baseline–pre supplementation) and T5 (the end of the study) separately, satisfactory descriptive and predictive parameters were obtained in the case of T5 dataset (6 components give R2X = 0.962 Q2 = 0.398) and suggested an interesting clustering of AB and BA SF samples in the t2/t6 scores plot (Fig 9).

**Fig 10:**
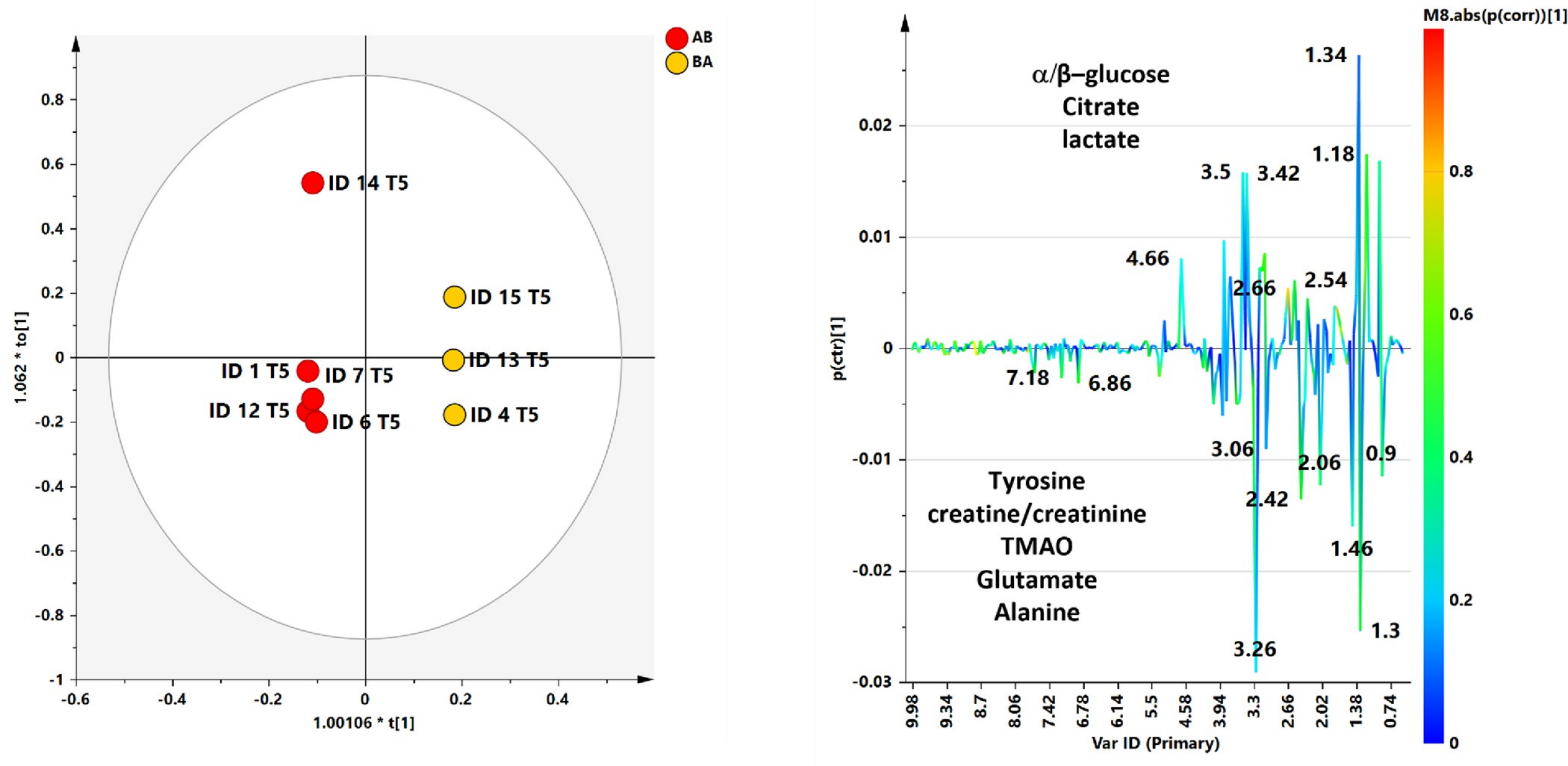
OPLS-DA t[1]/t[2] scores plot for AB and BA SF class samples. (b) S line plot for the model colored according to the correlation-scaled coefficient. The color bar indicates the correlation of the metabolites discriminating among classes.

Thus, the following OPLS-DA analysis enhanced the observed AB and BA clustering giving a model descripted by 1+3+0 components and good statistical parameters (R2X = 0.569, R2Y = 0.99, Q2 = 0.451) (Fig 10).

**Fig 11:**
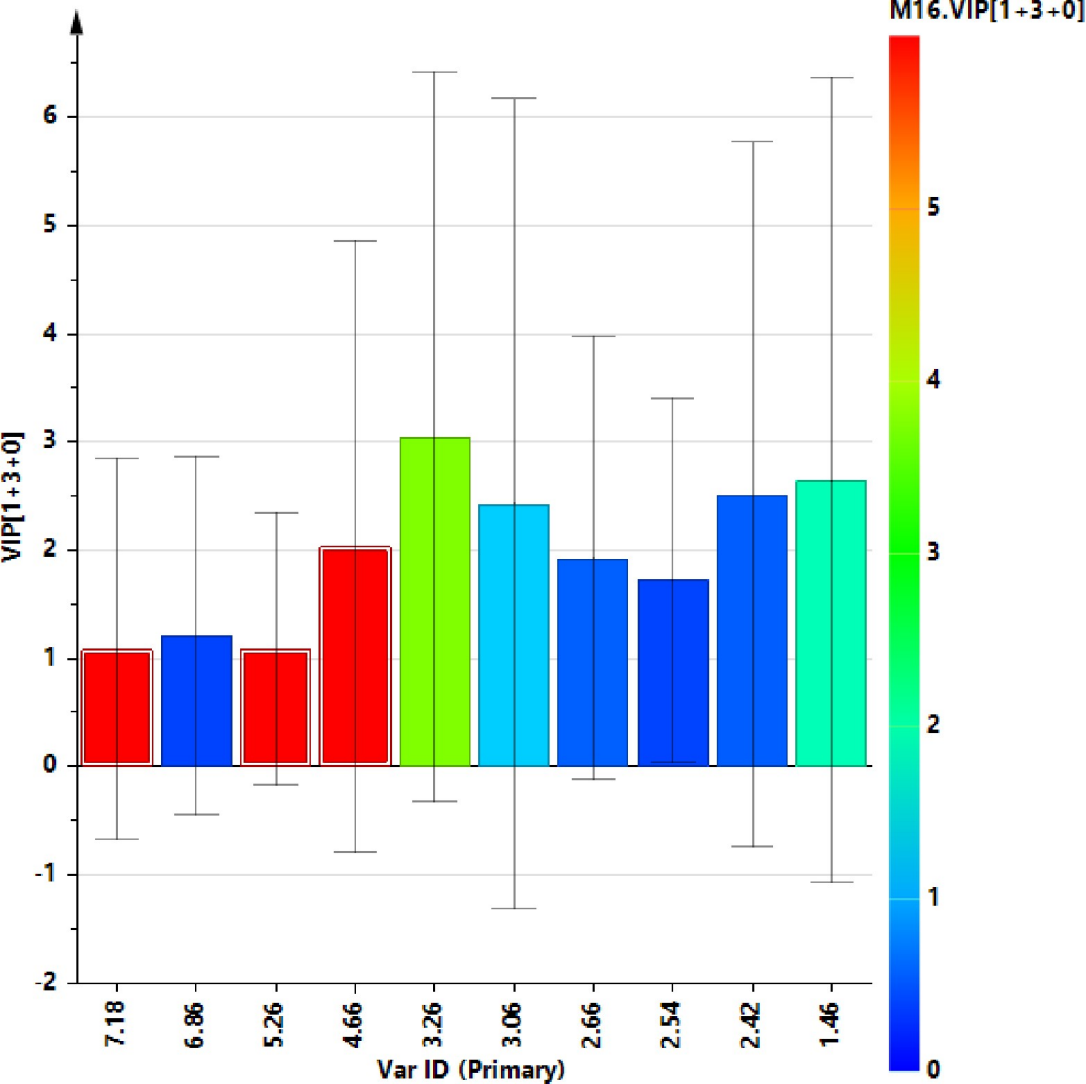
VIP plot displays the VIP values (≥1) as a column plot with jack-knife uncertainty bars (95%).

A total of 8 discriminating metabolites were identified form the relative S line plot and selected for strong discrimination power (VIP ≥1). A quantitative comparison of discriminating metabolites (Fig 9) between the two classes was performed by calculating the fold change (FC) ratio. Among selected metabolites with strong discrimination power no one reach statistical significance (*p* ≤ 0.05) (Fig 11).

A higher relative content of tyrosine (7.18, 6.86), creatine/creatinine (3.06), TMAO (3.26 ppm), glutamate/glutamine (2.42) and alanine (1.50) were observed in AB samples. On the contrary, BA samples have a higher relative content of a/β-glucose (4.66, 5.26), citrate (2.66, 2.54) and lactate (1.34) (Table 4).

**Table 4:** Fold change calculated as the ratios between the two group means (BA/AB). The significant features are beyond the given threshold (FC = 1).

Metabolites	Chemical shift (ppm)	Fold change ratio (BA/AB)	LOG2 FC
**tyrosine**	7.18	0.42718	-1.2271
**citrate**	2.54	1.553	0.63504
**glutamine/glutamate**	2.42	0.67191	-0.57366
**alanine**	1.46	0.71361	-0.48679
**creatine/creatinine**	3.06	0.75736	-0.40095
**TMAO**	3.26	0.88137	-0.18218
**a-glucose**	5.26	1.1323	0.17925
**b-glucose**	4.66	1.1102	0.15078

## Discussion

The primary clinical significance of our results suggests that the use of Flexadin Advanced® as a supplement over a period of eight weeks resulted in improvements in mobility, as assessed by owners in a population of dogs with mild to moderate OA after only four weeks of treatment. In contrast, its absence was associated with an increase in the risk of OA crisis and a decrease in the pain threshold of the most affected joint. Furthermore, the synovial fluid metabolic profile showed moderate differences between beginning and ending supplementation, with a particular influence associated with the timing of UCII®-BW administration. Clinical examination, gait analysis and activity assessment were not different between the supplemented and placebo groups.

The treatment of OA is typically confined to NSAIDs, but their usage is widely known to be associated with potential negative effects [37]. As a result, research into the efficacy of alternative therapies for the management of this chronic condition is required to improve the quality of companion animals’ life [38].

Nutritional supplements are widely accepted as part of a multimodal approach to manage osteoarthritis. Undenatured type collagen and Boswellia serrata are two oral supplements that are available in OA patients. In particular, undenatured type II collagen has been shown to provide significant clinical benefit in supporting canine joint health in both short-term (24,25) and long-term (23,27,29,30) studies. In addition, the use of Boswellia serrata in combination with other oral supplements has been shown to be helpful in reducing chronic pain, improving clinical symptoms (especially in severe cases), and reducing inflammatory biomarkers [19–22,39]. Thus, their combination should have potential additional effects in the management of canine OA.

Previous studies investigating the clinical effects of UCII® alone and in combination have shown interesting results after 30 days of treatment in terms of improvement in mobility, reduction in joint pain and improvement in synovial fluid metabolism [24,25].

In agreement with previous findings, the combination of UCII® and boswellia serrata in this study promoted an improvement in mobility, as evidenced by a significant and clinically effective reduction in the LOAD score (by about 20%) after only 4 weeks of supplementation. [40,41]. It is noteworthy that, although not statistically significant, pain and ROM scores tended to increase during the placebo administration and washout period in the study dogs.

It is noteworthy that there was no difference in terms of other clinical parameters, such as lameness and baropodometric assessment, between the placebo and the supplement. In this study, the GLS of the dogs was slightly less than 95% at baseline, indicating mild lameness on the target limb, which reached normal levels (>95%) during follow-up in both groups.

It is likely that the assessment of gait analysis was influenced by the characteristics of the study sample, which consisted primarily of medium sized dogs with mild to moderate levels of osteoarthritis and various joint locations of the disease. The study by Gupta et al. (30) proved that dogs supplemented with UCII® showed significant improvement in lameness, as measured by vertical force analysis, at 90 days with a peak at 150 days. Therefore, given the duration of our study, it is possible that the shorter duration of supplementation may have influenced our data. It is plausible that longer administration of undenatured UCII®-BW could produce similar results, given the mechanism of action of oral tolerance [42].

Data from the accelerometers were inconsistent and did not provide significant insight in our context, despite the fact that different periods (day and night) were examined as reported by Lascelles *et al*.[41]. This may be due to the limited number of cases evaluated and the numerous technical issues encountered during recording.

With respect to the Von Frey pain threshold assessment in our dogs, we observed a reduction in pain threshold during the 4-week visit in both the UCII® BW and placebo groups. However, the pain levels in the placebo group were significantly lower than those in the UCII®-BW group. In this scenario, the administration of UCII®-BW resulted in an alleviation of the pain condition. This finding is also supported by the recorded OA crisis and the need for rescue analgesia during placebo administration.

^1^H NMR is a promising option as it has been shown to be highly valuable in the assessment of various disease states as a potential diagnostic and prognostic tool [43,44]. Particularly, it is widely used in metabolomics studies to examine OA at a molecular level. Also our research group used this type of analysis to assess OA joint in dogs and horses [33,45].

In this study, seven metabolites were distinguished between T0 and T5. Initially, the dominant metabolites were lactate, glutamate, alanine and TMAO. Conversely, at the end glucose and lipids prevailed. A significant difference between the two time periods was observed in the quantification of lactate which was higher at T0. The significance of lactate in the OA joint is a subject of controversy. Some authors have considered it to be an inflammatory biomarker, that can discriminate between different inflammatory states in the joint, indicating a predominance of anaerobic metabolism [45–47]. On the contrary, others have suggested that it cannot be considered a reliable biomarker on its own, because of its presence also in healthy condition. In our previous study, we also observed elevated lactate levels in the SFs of healthy patients [33]. Thus, this finding should be treated with caution, but demonstrates a change in metabolism over time that may have been influenced by supplementation. Given the limited evaluation of metabolomics, solely at T0 and T5, and the lack of immediate supplementation, we further investigated T5 samples using data from dogs that received UCII^®^-BW in the first period of the study, as well as from dogs that were given the supplement in the second period of the study. Even if this analysis was only performed on a small number of samples, however, an intriguing and predictive model was observed, differentiating eight metabolites, including tyrosine creatinine/creatinine, TMAO, glutamate/glutamine, and alanine in dogs that first received the supplement and then the placebo (AB), whereas glucose, citrate, and lactate were found in dogs that first received the placebo and then the supplement (BA).

The majority of the metabolites discovered in this study have been previously described in literature [48–50]. The discovery of glutamine, alanine, creatinine, and TMAO simultaneously demonstrated an altered metabolism, indicated by a decrease in glycosaminoglycan (GAG), hyaline cartilage collagen, and proteoglycan (as shown by the increase in glucosamine and amino acid levels), an increase in oxidative stress and inflammation (as evidenced by the increase in TMAO levels), and muscle breakdown (as proven by the increased levels of creatine/creatinine). The prevalence of α/β glucose, citrate and lactate, indicated a shift in joint metabolism towards predominant glycolysis and glucose metabolism after supplementation [33,51–53].

Although the robustness of the methodology used in this study, it has many limitations. First, the number of dogs analysed, that was lower than the number previous considered, due to difficulties in the owners’ compliance and consequent loss of cases for delayed visits. However, we maintained the minimal number to assure the power calculation considered a priori. Second, the low number of Actical devices and related technical issue, that probably influenced the consistence of our data. Third, data from Von Frey test can be affected by the absence of specie-specific validation of this tool. It is in fact validated to use in rodents but not in dogs. Moreover, we did not consider the pain threshold for each dog on the same not affected joint, because it was not feasible. Thus, we were unaware about the subjective pain threshold on not painful site.

Finally, the difficulty to collect the synovial fluid samples for each dog, the presence of blood contamination, and the following technical issue during the analysis, allowed to analyse a low number of SFs, that might have affected our data. Moreover, for ethical reason and for the clinical nature of the study, the decision to analyse SFs only at two times of the study (at the beginning and at the end) might have affected the value of this assessment and its predictivity.

## Conclusion

The results of the study showed that Flexadin Advanced ® Boswellia administered for 8 weeks in dogs affected by natural occurring OA can improve the mobility impairment already after 4 weeks. The absence of its supplementation resulted in an evident worsening in pain condition and change in metabolomic status of the joint.

## Supporting information

S1 Data (XLSX)
